# External Validation of Toulouse-Rangueil eGFR12 Prediction Model After Living Donor Nephrectomy

**DOI:** 10.3389/ti.2023.11619

**Published:** 2023-09-07

**Authors:** Suhani S. Patel, Bonnie E. Lonze, Teresa Po-Yu Chiang, Fawaz Al Ammary, Dorry L. Segev, Allan B. Massie

**Affiliations:** ^1^ Department of Surgery, Transplant Institute, NYU Langone Health, New York, NY, United States; ^2^ School of Medicine, University of California, Irvine, Irvine, CA, United States; ^3^ Scientific Registry of Transplant Recipients, Minneapolis, MN, United States

**Keywords:** external validation, predictive model, living donor renal function, kidney transplantation, chronic kidney disease

## Abstract

Decreased postdonation eGFR is associated with a higher risk of ESRD after living kidney donation, even when accounting for predonation characteristics. The Toulouse-Rangueil model (TRM) estimates 12 month postdonation eGFR (eGFR12) to inform counseling of candidates for living donation. The TRM was validated in several single-center European cohorts but has not been validated in US donors. We assessed the TRM in living kidney donors in the US using SRTR data 1/2000–6/2021. We compared the 2021 CKD-EPI equation eGFR12 observed estimates to the TRM eGFR12 predictions. Median (IQR) bias was −3.4 (−9.3, 3.4) mL/min/1.73 m^2^. Bias was higher for males vs. females (bias [IQR] −4.4 [−9.9, 1.8] vs. −2.9 [−8.8, 4.1]) and younger (31–40) vs. older donors (>50) (bias −4.9 [−10.6, 3.0] vs. −2.1 [−7.5, 4.0]). Bias was also larger for Black vs. White donors (bias (−6.7 [−12.1, −0.3], *p* < 0.001) vs. (−3.4 [−9.1, 3.1], *p* < 0.001)). Overall correlation was 0.71. In a sensitivity analysis using the 2009 CKD-EPI equation, results were generally consistent with exception to a higher overall bias (bias −4.2 [−9.8, 2.4]). The TRM overestimates postdonation renal function among US donors. Overestimation was greatest for those at higher risk for postdonation ESRD including male, Black, and younger donors. A new equation is needed to estimate postdonation renal function.

## Graphical Abstract

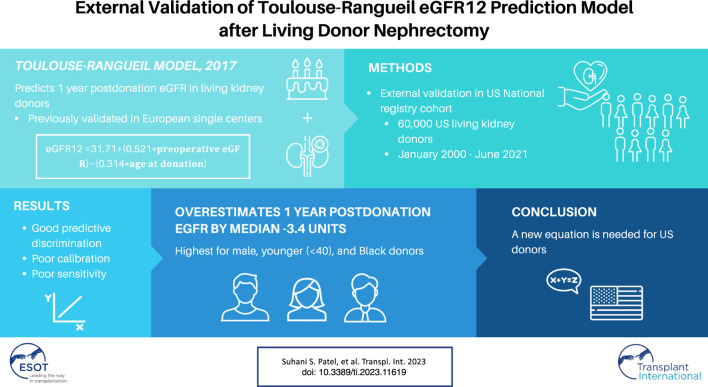

## Introduction

Although most living kidney donors (LKDs) do not experience renal complications, they face an increased long-term risk of end stage renal disease (ESRD) compared to healthy nondonors [1, 2]. A study of national registry data from the United States reported that estimated glomerular filtration rate (eGFR) at 6 months postdonation is associated with ESRD risk in LKDs (28% increased risk per 10 mL/min/1.73 m^2^), even after accounting for predonation characteristics [3]. Male donors, Black donors, and donors with a first-degree biological relationship to the recipient are at increased risk for ESRD postdonation [4]. A model to predict postdonation eGFR as a marker for risk of ESRD can aid in predonation donor evaluation and counseling.

The Toulouse-Rangueil model (TRM), developed by Benoit et al., estimates postdonation 12 month (eGFR12) based on predonation characteristics [5]. This prediction model was created using data from 133 LKDs from 2006 to 2014 in a single-center French cohort [5]. The final model included age at donation and predonation eGFR [5]. The authors reported a Pearson correlation of 0.65 (*p* < 0.001) and an area under the receiver operating curve (AUROC) of 0.83 (*p* < 0.001) in a validation cohort [5]. Subsequent studies externally validated the TRM in single-center cohorts in France (N = 400) [6], Portugal (N = 333) [7], and Germany (N = 130) [8]. All participants in the French and Portuguese cohort were White, and the racial composition of the German cohort is unknown [6–8]. These three cohorts demonstrated similar and moderately strong Pearson correlations (0.66/0.67/0.59) and AUROCs (0.86/0.83/0.87) suggesting validity in Western European populations [6–8].

However, applicability of the TRM to donors outside of Europe is unclear. To address this knowledge gap, we conducted a retrospective study to validate the TRM using national registry data from the United States.

## Materials and Methods

### Study Population

This study used data from the Scientific Registry of Transplant Recipients (SRTR). The SRTR data system includes data on all donor, wait-listed candidates, and transplant recipients in the US, submitted by the members of the Organ Procurement and Transplantation Network (OPTN). The Health Resources and Services Administration (HRSA), U.S. Department of Health and Human Services provides oversight to the activities of the OPTN and SRTR contractors. This dataset has previously been described elsewhere [9]. This study of deidentified data was determined to be “exempt: not human subjects research” by the institutional review board of NYU Langone (ID: i22-00146).

The study population included adult (age≥18) LKDs from 1 January 2000 to 2 June 2021. To remove erroneous datapoints, individuals with a predonation creatinine level outside of the range of 0.2–1.5 (N = 276), a predonation eGFR of less than 40 (N = 3), a 12 month postdonation creatinine outside of the range of 0.2–1.9 (N = 344), or an eGFR12 greater than 120 (N = 340) were excluded. Furthermore, individuals with a creatinine lower than or eGFR greater than their pre-donation levels were not included in the analysis. Domino and therapeutic donors were also excluded. The 12 month follow-up occurred between 9 and 18 months after donation.

### Validation of TRM

We compared the TRM eGFR12 predictions to the 2021 CKD-EPI creatinine equation eGFR12 observed estimates among LKDs using the following equation for TRM: $eGFR12\;\left(ml/\min/1.73\;m^{2}\right)=31.71+\left(0.521*preoperative\;eGFR\;\left(ml/\min\right)\right)-\left(0.314*age\;at\;donation\;\left(years\right)\right)$ [5]. We analyzed the bias (observed - predicted) and the Pearson correlation overall and in the following subgroups: gender, age, race (White/Black/Hispanic/Asian/Other), and relationship to the recipient (biological/non biological/non directed) to assess the validity of the proposed prediction model. We compared observed vs. predicted estimates using pooled t-tests. eGFR12 was binarized as < 60 vs. ≥ 60 mL/min/1.73 m^2^ to calculate the sensitivity, specificity, positive predictive value, and negative predictive value. We utilized the Hosmer-Lemeshow test to examine the model’s calibration. We constructed a histogram to examine the distribution of bias (observed-predicted). To assess the agreement, we created a Bland-Altman plot.

### Sensitivity Analysis

We conducted a sensitivity analysis in which we replicated the analysis using the older 2009 CKD-EPI creatinine equation, which estimates eGFR based on serum creatinine, age, sex, and race/ethnicity (coded as Black vs. non-Black) [10].

### Statistical Analysis

An α of 0.05 was considered statistically significant and all tests were two-sided. All analyses were performed using SAS (v.9.4) or R Studio (v.4.0.3).

## Results

### Study Population

The study population consisted of 60,839 LKDs from 2000 to 2021 (Table 1). Donors were predominantly female (64.1%) and White (72.4%) with a median age of 44 (Table 1). About 22.4% of LKDs have a history of smoking and 4.0% of LKDs have a history of hypertension (Table 1). 95.7% of donors had a predonation eGFR between 70–130 and 92.6% of donors had an eGFR12 between 30–90 (Table 1). The 12 month postdonation follow-up occurred between 9 and 18 months (median [IQR] 12.2 [11.8, 13.0]); 91% of follow-up occurred between 10 and 14 months.

**TABLE 1:** Kidney donor characteristics.

Donor characteristic		Entire sample (N = 60,839)
Gender, (%) Male		35.9
Age, median (IQR)		44 (34–53)
Race, (%)		
White		72.4
Black		9.1
Hispanic		13.4
Asian		3.8
Other		1.4
Predonation eGFR, (%)		
≥30–<50		0.03
≥50–<70		3.2
≥70–<90		26.0
≥90–<110		44.1
≥110–<130		25.6
≥130		1.2
eGFR12, (%)		
<30		0.005
≥30–<50		9.6
≥50–<70		52.6
≥70–<90		30.4
≥90–<110		6.6
≥110–<130		0.7
Laterality, (%)		
Left kidney		88.3
Right kidney		11.7
Procedure type, (%)		
Transabdominal		1.1
Flank (retroperitoneal)		4.3
Laparoscopic Not Hand-assisted		32.6
Laparoscopic Hand-assisted		58.7
Laparoscopic Unknown (inactive)		3.4
Natural Orifice		0.002
BMI, median (IQR)		26.6 (23.8, 29.6)
History of smoking, (%)		22.4
History of hypertension, (%)		4.0

eGFR12, Postdonation 12 month estimated glomerular filtration rate.

### Validation of TRM

Median bias [IQR] calculated as the difference between the observed estimate from the CKD-EPI equation and the predicted TRM eGFR12 was −3.4 [−9.3, 3.4] mL/min/ 1.73 m^2^ and mean bias was −2.5 mL/min/1.73 m^2^ (Table 2). Median bias was higher for all predicted vs. observed values. Furthermore, predicted values were statistically significantly different from observed values for all gender, age, and donor’s relationship to recipient subcategories (*p* < 0.001) (Table 2). Bias was higher for males vs. females (bias [IQR] −4.4 [−9.9, 1.8] vs. −2.9 [−8.8, 4.1]) and younger (31–40) vs. older donors (>50) (bias −4.9 [−10.6, 3.0] vs. −2.1 [−7.5, 4.0]) (Table 2). Bias was larger for Black vs. White donors (bias −6.7 [−12.1, −0.3] vs. −3.4 [−9.1, 3.1]) but lower for Asian and Hispanic donors compared to White donors (bias −1.3 [−8.5, 6.1], −1.4 [−8.1, 6.4] vs. −3.4 [-9.1, 3.1]) (Table 2).

**TABLE 2:** Median (IQR) bias and correlation overall and by subgroups.

Donor characteristic	Observed	Predicted	Bias	r	N	*p*-value
Overall	65.5 (56.7, 75.8)	69.5 (62.1, 77.5)	**−3.4 (−9.3, 3.4)**	0.71	60,839	<0.001
Gender						
Female	65.8 (57.0, 76.3)	69.5 (61.9, 77.2)	**−2.9 (−8.8, 4.1)**	0.71	38,992	<0.001
Male	64.8 (56.2, 75.0)	69.6 (62.5, 77.7)	**−4.4 (−9.9, 1.8)**	0.72	21,847	<0.001
Age						
18–30	77.7 (69.3, 88.2)	84.4 (76.5, 88.2)	**−4.0 (−11.0, 3.3)**	0.58	10,028	<0.001
31–40	70.3 (61.7, 79.9)	76.2 (69.5, 81.1)	**−4.9 (−10.6, 3.0)**	0.61	14,714	<0.001
41–50	63.5 (56.4, 71.9)	68.5 (62.8, 74.2)	**−3.7 (−9.5, 3.2)**	0.60	17,238	<0.001
>50	58.2 (51.3, 65.8)	61.3 (55.6, 66.5)	**−2.1 (−7.5, 4.0)**	0.61	18,859	<0.001
Race						
White	64.2 (55.8, 74.0)	68.2 (60.9, 75.6)	**−3.4 (−9.1, 3.1)**	0.70	44,016	<0.001
Black	62.4 (54.1, 72.3)	69.6 (62.1, 77.3)	**−6.7 (−12.1, −0.3)**	0.70	5,516	<0.001
Hispanic	73.6 (63.9, 85.3)	76.2 (68.7, 83.0)	−1.4 (−8.1, 6.4)	0.67	8,130	0.26
Asian	71.2 (61.7, 82.1)	73.4 (66.3, 80.7)	−1.3 (−8.5, 6.1)	0.66	2,328	0.16
Other	67.2 (58.5, 77.2)	72.2 (64.4, 79.7)	**−4.4 (−10.7, 2.9)**	0.66	849	<0.001
Relationship to recipient						
Biological	66.9 (57.8, 77.7)	71.2 (63.4, 79.0)	**−3.6 (−9.6, 3.5)**	0.70	28,708	<0.001
Non-biological	64.2 (55.9, 74.4)	68.2 (61.0, 75.6)	**−3.3 (−9.0, 3.4)**	0.71	26,052	<0.001
Non-directed	64.3 (55.8, 74.4)	68.3 (61.1, 76.0)	**−3.4 (−9.1, 3.2)**	0.72	6,078	<0.001

r, correlation.

Overall bias (observed-predicted) −3.4 mL/min/1.73 m^2^ and correlation 0.71. All predicted values are statistically significantly different from observed with exception to Hispanic and Asian donors.

Bold values indicate statistical significance defined as *p* < 0.05.

**FIGURE 1:**
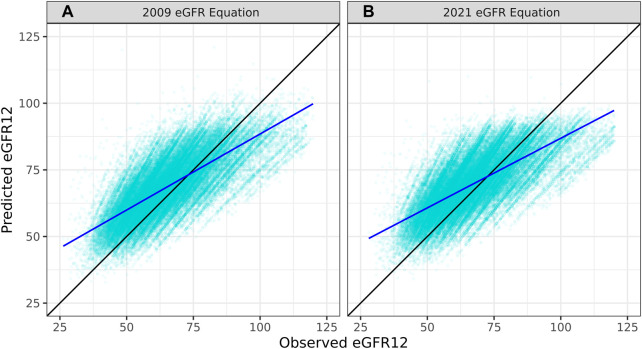
Pearson correlation between predicted eGFR12 and observed eGFR12 by CKD-EPI equation (eGFR: mL/min/1.73 m^2^) **(A)** using the 2009 eGFR equation (r = 0.71, *p* < 0.001) **(B)** using the 2021 eGFR equation (B: r = 0.74, *p* < 0.001) *eGFR12, Postdonation 12 month estimated glomerular filtration rate.

**FIGURE 2:**
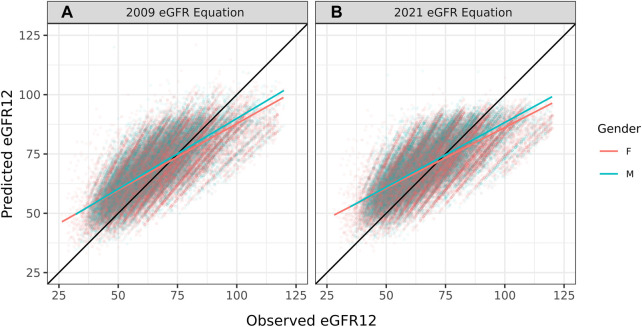
Pearson correlation between predicted eGFR12 and observed eGFR12 by CKD-EPI equation (eGFR: mL/min/1.73 m^2^). **(A)** using the 2009 eGFR equation, stratified by gender **(B)** using the 2021 eGFR equation, stratified by gender *eGFR12, Postdonation 12 month estimated glomerular filtration rate.

**FIGURE 3:**
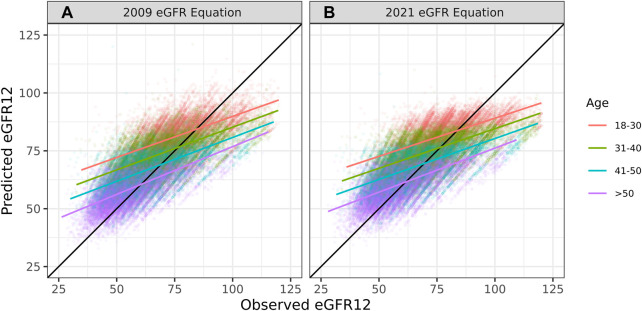
Pearson correlation between predicted eGFR12 and observed eGFR12 by CKD-EPI equation (eGFR: mL/min/1.73 m^2^). **(A)** using the 2009 eGFR equation, stratified by age **(B)** using the 2021 eGFR equation, stratified by age *eGFR12, Postdonation 12 month estimated glomerular filtration rate.

**FIGURE 4:**
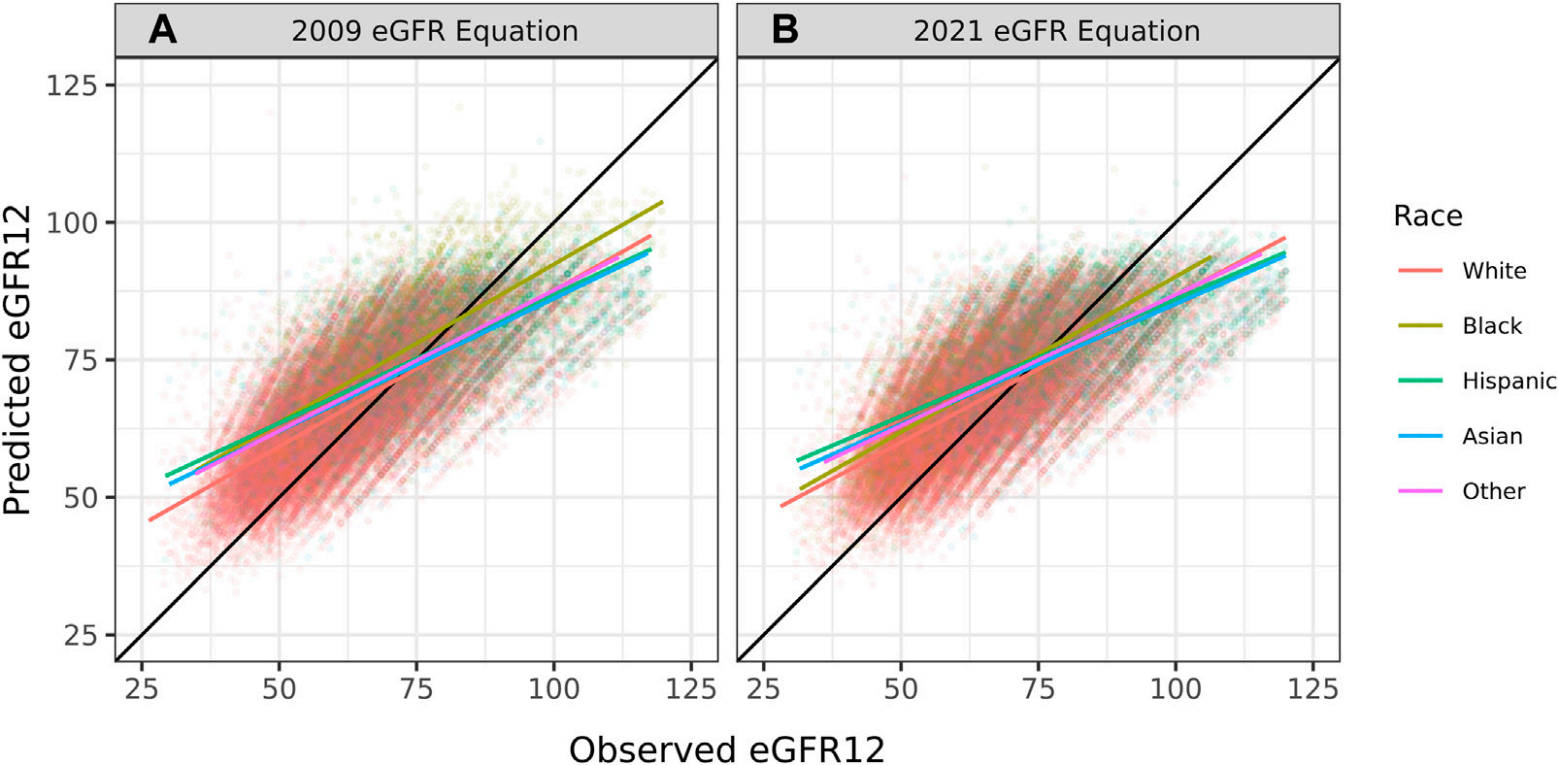
Pearson correlation between predicted eGFR12 and observed eGFR12 by CKD-EPI equation (eGFR: mL/min/1.73 m^2^). **(A)** using the 2009 eGFR , stratified by race **(B)** using the 2021 eGFR equation, stratified by race *eGFR12, Postdonation 12 month estimated glomerular filtration rate.

The overall correlation between TRM predicted and observed values was 0.71 (Table 2; Figure 1B). Moderately strong correlations exist among gender and donor’s relationship to recipient subcategories (correlation (corr.) range 0.70–0.72) (Table 2; Figure 2B). Correlations by age ranged from 0.58 to 0.61; the lowest correlation among all subgroups was donors aged 18–30 (Table 2; Figure 3B). Lower age was associated with larger overestimation of eGFR12 (Figure 3). Asian and Hispanic donors had marginally lower correlations vs. White donors (corr. 0.66, 0.67 vs. 0.70) (Table 2; Figure 4B).

**FIGURE 5:**
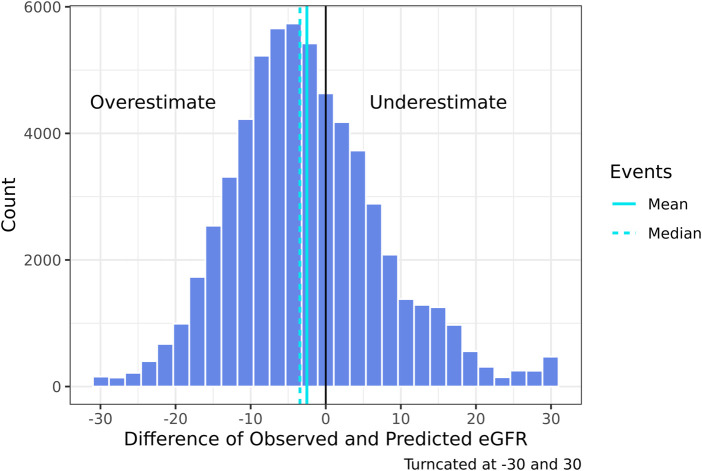
Histogram of the difference of 2021 CKD-EPI observed—predicted eGFR12 (mean: −2.51) (median: −3.44) (eGFR: mL/min/1.73 m^2^) *eGFR12, Postdonation 12 month estimated glomerular filtration rate.

**FIGURE 6:**
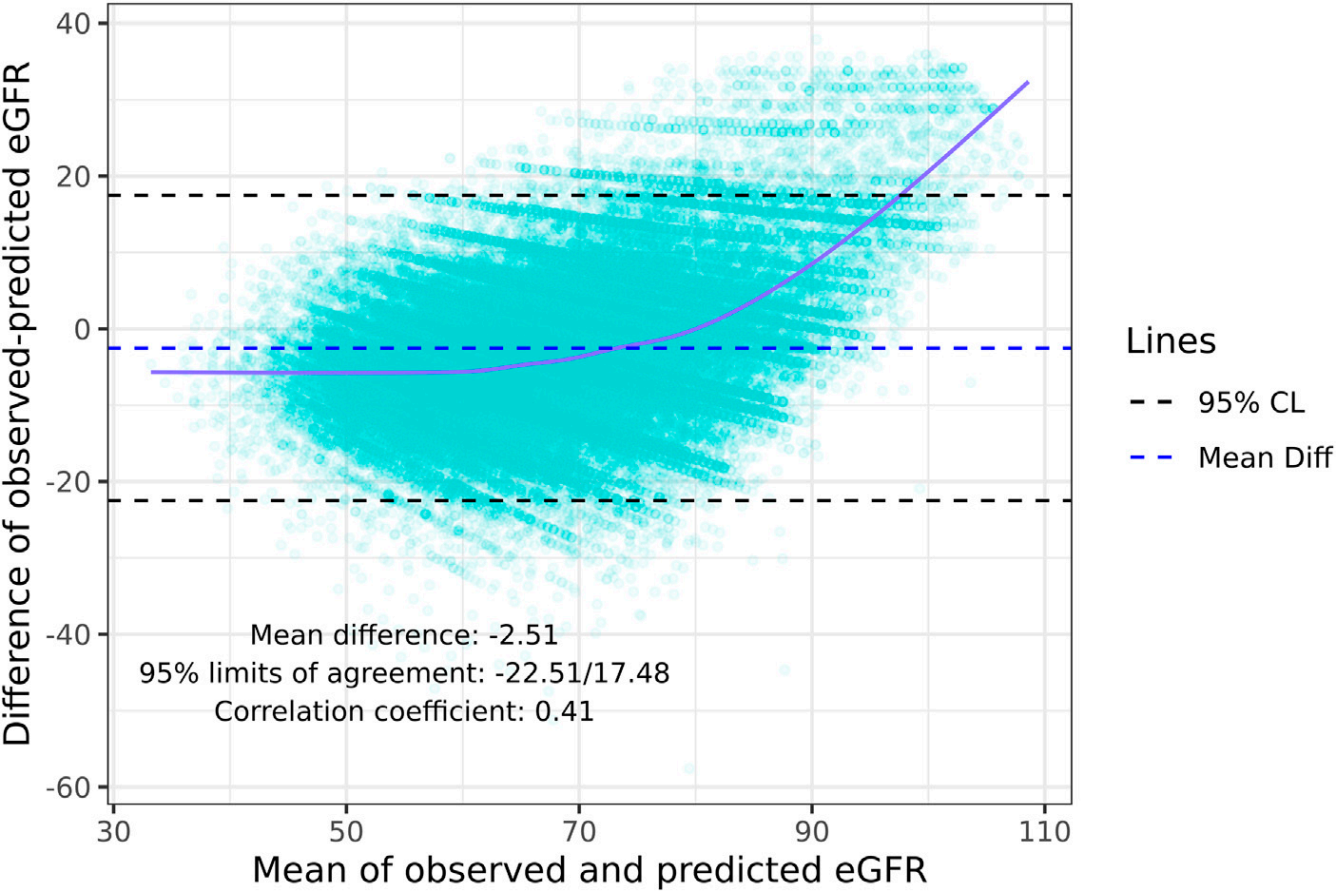
Bland-Altman plot: Agreement and correlation coefficient between the difference and mean of the predicted eGFR12 and observed 2021 CKD-EPI eGFR12 (eGFR: mL/min/1.73 m^2^). *eGFR12, Postdonation 12 month estimated glomerular filtration rate.

Although the specificity was large (0.94), the sensitivity was low (0.45) demonstrating a poor ability to estimate LKDs with <60 eGFR (Table 3). Among donors predicted to have ≥60 eGFR12, 77% had an observed eGFR12 ≥60; among donors predicted to have a <60 eGFR12, 80% had an observed eGFR12 <60 (Table 3). According to the Hosmer-Lemeshow test, the model had good fit (*p* = 0.07) (Table 4). The mean bias (observed-predicted) is lower than the median bias (−2.51 vs. −3.44) (Figure 5). According to the Bland-Altman plot, the 95% limit of agreement is −22.51/17.48 (Figure 6).

**TABLE 3:** Contingency table to summarize the relationship between predicted and observed eGFR12 < 60 mL/min/1.73 m^2^.

		Observed eGFR12		Total
		<60	≥60	
Predicted eGFR12	<60, n (%)	9,286 (44.7)	2,258 (5.6)	11,544
	≥60, n (%)	11,509 (55.3)	37,786 (94.4)	49,295
Total		20,795	40,044	60,839

eGFR12, Postdonation 12 month estimated glomerular filtration rate.

Sensitivity 0.45, specificity 0.94, positive predictive value 0.80, negative predictive value 0.77.

**TABLE 4:** Hosmer-Lemeshow test for goodness of fit, *p* = 0.07.

Group	Total	eGFR12 < 60		eGFR12 ≥ 60	
		Obs	Exp	Obs	Exp
1	6,049	76	90.9	5,973	5,958.1
2	6,080	234	257.8	5,846	5,822.2
3	6,111	540	514.0	5,571	5,597.0
4	6,083	842	868.8	5,241	5,214.2
5	6,040	1,333	1,327.5	4,707	4,712.5
6	6,077	1,995	1914.4	4,082	4,162.6
7	6,095	2,607	2,632.0	3,488	3,463.0
8	6,024	3,391	3,414.5	2,633	2,609.5
9	6,103	4,389	4,360.7	1,714	1,742.3
10	6,177	5,388	5,414.7	789	762.3
$\chi^{2}$	14.7				
*p*-value	0.07				

Obs, observed; Exp, expected; eGFR12, Postdonation 12 month estimated glomerular filtration rate.

### Sensitivity Analysis

In a sensitivity analysis using the 2009 CKD-EPI eGFR equation, results were generally consistent with our main analysis. 2009 CKD-EPI estimates of predonation eGFR ≥90 and eGFR12 ≥ 70 were slightly lower than 2021 CKD-EPI estimates (Supplementary Table S1; Table 1). Compared to the 2021 CKD-EPI based TRM predictions, the 2009 CKD-EPI overall median bias was higher (median [IQR] −4.2 [−9.8, 2.4] vs. −3.4 [−9.3, 3.4]) (Supplementary Table S2; Table 2). Median bias was higher for younger (31–40) vs. older donors (>50) (median −5.4 [−11.1, 2.2] vs. −2.9 [−8.1, 2.8]), and males vs. females (median −5.2 [−10.5, 0.7] vs. −3.6 [−9.4, 3.2]) (Supplementary Table S2). While bias was still higher for Black vs. White donors (median −5.6 [−11.7, 1.4] vs. −4.3 [−9.7, 2.0]), bias based on the 2009 CKD-EPI for Black donors was slightly lower than the bias based on the 2021 CKD-EPI (median −5.6 [−11.7, 1.4] vs. −6.7 [−12.1, −0.3]). The overall correlation based on the 2009 CKD-EPI estimates was slightly larger than the 2021 CKD-EPI based correlation (0.74 vs. 0.71) (Supplementary Table S2; Table 2; Figure 1A). The specificity was the same (0.94) and the 2009 CKD-EPI based sensitivity was slightly larger but comparable to the 2021 CKD-EPI based sensitivity (0.50 vs. 0.45) (Supplementary Table S3; Table 3). While the 2009 CKD-EPI based TRM estimates failed the Hosmer-Lemeshow test for model fit (*p* < 0.001), the 2021 CKD-EPI based TRM estimates passed the Hosmer-Lemeshow test (*p* = 0.07) (Supplementary Table S4; Table 4). The median bias (observed-predicted) is larger than the mean bias (−4.16 vs. −3.33) (Supplementary Figure S1). The 95% limit of agreement is -22.72/16.06 according to the Bland-Altman plot (Supplementary Figure S2).

## Discussion

In this external validation study, the TRM had good discrimination but poor calibration in predicting eGFR12 postdonation in a national registry cohort from the United States. Correlation between observed and predicted eGFR12 in the US cohort was moderately strong with a correlation coefficient of 0.71; higher than in previous external validation cohorts in France, Portugal, and Germany [6–8]. However, the TRM demonstrated bias, overestimating eGFR12 by median 3.4 units; the bias was more pronounced for male donors, younger donors (<40), and Black donors, populations at higher long-term risk for ESRD [4]. Moreover, the TRM performed poorly in predicting the binary outcome of eGFR12<60; specificity was high at 94%, but sensitivity was only 45%. As such, the TRM will fail to identify many donors at risk of poor postdonation renal function. Therefore, we recommend that the TRM not be used for evaluation of candidates for living kidney donation in the United States. Moreover, the TRM should be used with caution outside of Europe, and may be inappropriate for younger donor candidates or nonwhite donor candidates in Europe.

While there is currently a lack of global consensus on a universal eGFR equation, serum creatinine based eGFR equations are the most widely used [11]. The TRM model was developed and externally validated in France [6] and Portugal [7] using the serum creatinine based 2009 eGFR CKD-EPI equation although the German external validation paper [8] uses the Modification of Diet in Renal Disease (MDRD) eGFR equation. A retrospective analysis found that the 2009 CKD-EPI eGFR equation has higher accuracy than the MDRD equation when compared to the gold standard of GFR measured through the clearance of exogenous filtration markers [12]. Since the creation of the TRM, a new race-free 2021 CKD-EPI equation has been developed. This equation is recommended by the National Kidney Foundation and is widely utilized by US clinicians. However, according to Husain et al., the 2021 race-free CKD-EPI eGFR equation increases estimates overall by 2.1 mL/min/1.73 m^2^ (IQR 0.0–3.3) and decreases estimates by 12.9 mL/min/1.73 m^2^ (IQR 17.2–9.8) among Black donors [13]. Augustine et al. similarly found that among Black donors, the 2021 CKD-EPI equation underestimates eGFR but that the cystatin C based 2021 equation performed better [14]. Our postdonation estimation may be improved with the cystatin C based 2021 equation although the SRTR does not collect this metric. We chose to focus this study on the 2021 CKD-EPI equation eGFR estimates due to the availability of serum creatinine data and because it is the current standard of practice in pre-donation donor evaluation in the US.

According to our sensitivity analysis, the 2009 CKD-EPI equation based TRM predictions demonstrated a higher overall median bias compared to the 2021 CKD-EPI equation (2009 CKD-EPI: −4.2 vs. 2021 CKD-EPI: −3.4). Additionally, the Hosmer-Lemeshow test for model fit failed based on the 2009 CKD-EPI estimates but passed based on 2021 CKD-EPI estimates (2009 CKD-EPI: *p* < 0.001 vs. 2021 CKD-EPI: *p* = 0.07). Median bias was higher for Black vs. White donors (bias −5.6 vs. −4.3), younger (31–40) vs. older (>50) donors (bias −5.4 vs. −2.9), and male vs. female donors (bias −5.2 vs. −3.6). Overall, the TRM predictions based on the 2009 CKD-EPI eGFR estimates performed similarly and, in some cases, worse than the 2021 CKD-EPI eGFR based predictions. Irrespective of which equation is utilized, the TRM’s performance remains questionable and potentially problematic for the estimation of eGFR12 in US cohorts due to concerns over calibration and disparities in Black, younger, and male donors.

Although renal failure is rare among LKDs, there are two prominent studies that have indicated an association between living donor nephrectomy and ESRD compared to healthy nondonors [1, 2]. Because ESRD is an uncommon outcome, a proxy may aid in identifying candidate donors at higher risk. An analysis of 71,468 US LKDs reported a 28% increased chance of ESRD per 10 mL/min/1.73 m^2^ decrease in 6 month postdonation eGFR (eGFR6) after adjusting for age, race, sex, body mass index, and biological relationship [3]. There are several studies that indicate an association between predonation eGFR and postdonation ESRD risk [15, 16]. Prior research indicates that eGFR6 may fully mediate the association between predonation eGFR and ESRD.

Importantly, while early postdonation eGFR is a potential marker of long-term ESRD risk, it is only one component of full assessment of function of the remaining kidney following living donor nephrectomy. A prior registry study of living kidney donors in the United States reported that at the time of donation 3.2% of donors had hypertension and 0.05% of donors had diabetes [17]. One-year postdonation, incidence of *de novo* hypertension was 162/10,000 donors while incidence of diabetes was 6/10,000 [17]. Blood pressure, diabetes risk, and proteinuria should be carefully monitored in living kidney donors to ensure long-term renal health.

Our findings provide additional context to prior studies from single-center French, Portuguese, and German cohorts. The mean difference between observed-predicted (95% limit of agreement) was −2.5 (−22.5/17.5) compared to −2.4 (−23.1/18.3) in the French cohort [6] and +2.3 (−21.4/26.1) in the Portuguese cohort [7]. However, in our study, performance of the equation was worse for clinically important subgroups of younger donors and Black donors. Interestingly, the correlation between observed and predicted values was higher in our cohort (0.71) compared to these prior studies (0.66/0.67/0.59) [6–8]. While the French cohort demonstrated a higher sensitivity in predicting eGFR<60 (0.59 in the French cohort vs. 0.45 in our cohort) but lower specificity (0.89 in the French cohort vs. 0.94 in our cohort) [6], the Portuguese validation study reported a comparable sensitivity (0.47 in the Portuguese cohort vs. 0.45 in our cohort) and specificity (0.93 in the Portuguese cohort vs. 0.94 in our cohort) [7]. While our study population was 28% non-White, all donors in the French and Portuguese population were White. The lack of racial diversity in previous external validation studies necessitates the study of the TRM in more diverse European populations. Since our study population was larger and more heterogeneous than prior cohorts, caution may be warranted when interpreting the TRM even in European settings, particularly for younger donor candidates or racial/ethnic minorities, for whom the TRM had the highest bias in our study.

As noted in commentary by Wang and Gard, the original TRM risks bias from deriving the model from LKDs vs. candidates for LKD [18]. This bias affects our study as well, and is inherent to any study of postdonation renal function since postdonation renal function can only be assessed in individuals who actually undergo donor nephrectomy. Wang and Gard also noted that eGFR12 is an imperfect indicator of future ESRD risk [3, 18], although prior research has shown an association between early postdonation renal function and long-term ESRD risk [3]. If anything, these two concerns further weaken the case for clinical use of the TRM for evaluating LKD candidates in the United States.

Our findings must be interpreted in the context of the limitations of our study. Approximately 44% of living donors who were otherwise eligible for inclusion in our study did not have serum creatinine assessed at 12 months postdonation, and so were excluded from the analysis. However, we have no reason to think that the TRM would perform differently among donors who were lost to followup by the transplant center. Our study follow-up was not at exactly 12 months, but rather between 9 and 18 months postdonation. Having said that, 91% of samples were collected within 2 months of the 12 month follow-up date. Further, our larger sample size allowed us to conduct subgroup analysis, revealing varying levels of bias across racial, gender, and age subcategories. Future studies of the TRM in European cohorts should investigate potential bias within important demographic subgroups.

Taken as a whole, while the TRM had good predictive discrimination in an American cohort, it systematically overestimated postdonation renal function in this cohort. Notably, overestimation was greatest for those at higher risk for postdonation ESRD including male, Black, and younger donors. A new equation is needed to estimate postdonation renal function in LKDs in the United States. The TRM should be used with caution outside of Europe, and with younger donor candidates or nonwhite ethnic/racial minority candidates in Europe.

## Data Availability

Publicly available datasets were analyzed in this study. This data can be found here: https://www.srtr.org/requesting-srtr-data/data-requests/.

## Abbreviations

AUROC, Area under the receiver operating curve; CKD, Chronic kidney disease; CKD-EPI, Chronic Kidney Disease Epidemiology Collaboration; eGFR12, Postdonation 12-month estimated glomerular filtration rate; ESRD, End stage renal disease; HRSA, Health Resources and Services Administration; IQR, Interquartile range; LKD, Living kidney donor; MDRD, Modification of Diet in Renal Disease; OPTN, Organ Procurement and Transplantation Network; SRTR, Scientific Registry of Transplant Recipients; TRM, Toulouse-Rangueil model.
