# Supplementary material for: External Validation of Toulouse-Rangueil eGFR12 Prediction Model After Living Donor Nephrectomy
Source: Transpl Int. 2023 Sep 7;36:11619. doi: 10.3389/ti.2023.11619 (PMC10511758; doi:10.3389/ti.2023.11619)
Supplement: Supplementary file 1 [file DataSheet1.docx]

**Supplementary Table S1. Kidney Donor Characteristics.** eGFR calculated by 2009 CKD-EPI Equation.

| Donor Characteristic | | Entire Sample (N=60839) |
| --- | --- | --- |
| Predonation eGFR, (%) | |  |
|  | ≥ 30 - < 50 | 0.1 |
|  | ≥ 50 - < 70 | 4.9 |
|  | ≥ 70 - < 90 | 29.5 |
|  | ≥ 90 - < 110 | 42.2 |
|  | ≥ 110 - < 130 | 21.0 |
|  | ≥ 130 | 2.4 |
| eGFR12, (%) | |  |
|  | < 30 | 0.03 |
|  | ≥ 30 - < 50 | 13.3 |
|  | ≥ 50 - < 70 | 53.3 |
|  | ≥ 70 - < 90 | 27.0 |
|  | ≥ 90 - < 110 | 5.9 |
|  | ≥ 110 - < 130 | 0.6 |

*eGFR12 = Postdonation 12-month estimated glomerular filtration rate

**Supplementary Table S2. Median (IQR) bias and correlation overall and by subgroups.** Overall bias (observed-predicted) -4.2 mL/min/1.73m^2^ and correlation 0.74. All predicted values are statistically significantly different from observed. eGFR calculated by 2009 CKD-EPI Equation.

| Donor Characteristic | | Observed | Predicted | Bias | r | N | P-Value |
| --- | --- | --- | --- | --- | --- | --- | --- |
| Overall | | 63.5 (54.7,74.0) | 68.2 (60.5,76.6) | **-4.2 (-9.8,2.4)** | 0.74 | 60839 | <0.001 |
| Gender | |  |  |  |  |  |  |
|  | Female | 63.9 (55.1,74.7) | 68.1 (60.3,76.5) | **-3.6 (-9.4,3.2)** | 0.73 | 38992 | <0.001 |
|  | Male | 62.7 (54.2,73.0) | 68.3 (60.8,76.7) | **-5.2 (-10.5,0.7)** | 0.75 | 21847 | <0.001 |
| Age | |  |  |  |  |  |  |
|  | 18-30 | 76.9 (68.2,87.3) | 83.5 (76.0,88.0) | **-4.8 (-11.6,2.8)** | 0.59 | 10028 | <0.001 |
|  | 31-40 | 69.1 (60.7,78.6) | 75.6 (68.5,80.1) | **-5.4 (-11.1,2.2)** | 0.61 | 14714 | <0.001 |
|  | 41-50 | 61.4 (54.5,69.7) | 67.3 (61.4,72.7) | **-4.4 (-10.1,2.0)** | 0.61 | 17238 | <0.001 |
|  | >50 | 55.4 (48.8,62.9) | 59.2 (53.7,64.6) | **-2.9 (-8.1,2.8)** | 0.62 | 18859 | <0.001 |
| Race | |  |  |  |  |  |  |
|  | White | 61.4 (53.1,71.2) | 66.1 (59.0,73.9) | **-4.3 (-9.7,2.0)** | 0.72 | 44016 | <0.001 |
|  | Black | 69.4 (59.9,80.9) | 75.6 (66.9,84.5) | **-5.6 (-11.7,1.4)** | 0.72 | 5516 | <0.001 |
|  | Hispanic | 70.9 (61.3,82.3) | 74.3 (66.7,81.8) | **-2.5 (-9.1,4.8)** | 0.69 | 8130 | <0.001 |
|  | Asian | 68.3 (58.8,79.3) | 71.7 (64.2,79.3) | **-2.6 (-9.3,4.6)** | 0.69 | 2328 | <0.001 |
|  | Other | 64.7 (55.8,74.2) | 70.4 (62.6,78.1) | **-5.3 (-11.4,1.8)** | 0.68 | 849 | <0.001 |
| Relationship to recipient | |  |  |  |  |  |  |
|  | Biological | 65.4 (56.1,76.2) | 70.1 (62.1,78.6) | **-4.3 (-10.1,2.6)** | 0.73 | 28708 | <0.001 |
|  | Non-biological | 62.0 (53.6,72.1) | 66.5 (59.3,74.5) | **-4.1 (-9.6,2.3)** | 0.73 | 26052 | <0.001 |
|  | Non-directed | 62.1 (53.7,72.1) | 66.8 (59.3,75.2) | **-4.2 (-9.7,2.1)** | 0.75 | 6078 | <0.001 |

*r = correlation

**Supplementary Table S3. Contingency table to summarize the relationship between predicted and observed eGFR12 < 60 mL/min/1.73m^2^.** Sensitivity 0.50, specificity 0.94, positive predictive value 0.84, negative predictive value 0.74. eGFR calculated with 2009 CKD-EPI Equation.

|  | | Observed eGFR12 | | Total |
| --- | --- | --- | --- | --- |
|  |  | **< 60** | **≥ 60** |  |
| Predicted eGFR12 | < 60, n (%) | 11996 (49.5) | 2326 (6.4) | 14322 |
|  | ≥ 60, n (%) | 12222 (50.5) | 34295 (93.6) | 46517 |
| Total | | 24218 | 36621 | 60839 |

*eGFR12 = Postdonation 12-month estimated glomerular filtration rate

**Supplementary Table S4. Hosmer-Lemeshow test for goodness of fit, p<0.001.** eGFR calculated with 2009 CKD-EPI Equation.

| Group | Total | eGFR12 < 60 | | eGFR12 ≥ 60 | |
| --- | --- | --- | --- | --- | --- |
|  |  | **Obs** | **Exp** | **Obs** | **Exp** |
| 1 | 6091 | 96 | 98.6 | 5995 | 5992.4 |
| 2 | 6085 | 330 | 312.0 | 5755 | 5773.0 |
| 3 | 6079 | 611 | 648.8 | 5468 | 5430.2 |
| 4 | 6075 | 1128 | 1129.0 | 4947 | 4946.0 |
| 5 | 6088 | 1883 | 1747.6 | 4205 | 4340.4 |
| 6 | 6129 | 2391 | 2492.8 | 3738 | 3636.2 |
| 7 | 6065 | 3234 | 3262.1 | 2831 | 2802.9 |
| 8 | 6077 | 4110 | 4083.5 | 1967 | 1993.6 |
| 9 | 6092 | 4859 | 4871.1 | 1233 | 1220.9 |
| 10 | 6058 | 5576 | 5572.9 | 482 | 485.1 |
| $\boldsymbol{\chi}^{\boldsymbol{2}}$ | 26.6 | | | | |
| P-value | <0.001 | | | | |

*Obs = observed

*Exp = expected

*eGFR12 = Postdonation 12-month estimated glomerular filtration rate

**Supplementary Figure 1. Histogram of the difference of 2009 CKD-EPI observed - predicted eGFR12 (mean: -3.33) (median: -4.16) (eGFR: mL/min/1.73m^2^).**

**
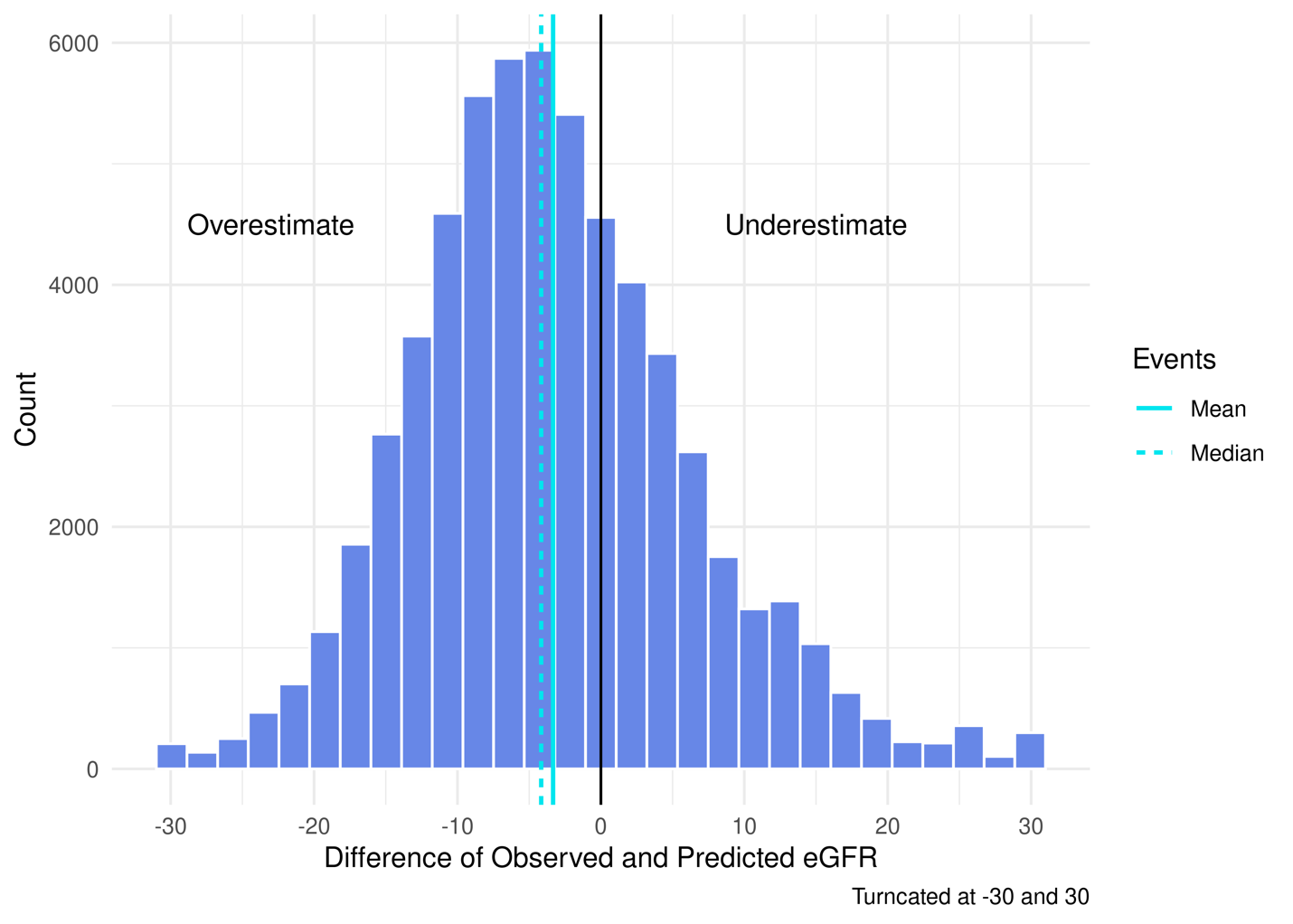
**

*eGFR12 = Postdonation 12-month estimated glomerular filtration rate

**Supplementary Figure 2. Bland-Altman plot: Agreement and correlation coefficient between the difference and mean of the predicted eGFR12 and observed 2009 CKD-EPI eGFR12 (eGFR: mL/min/1.73m^2^).**

**
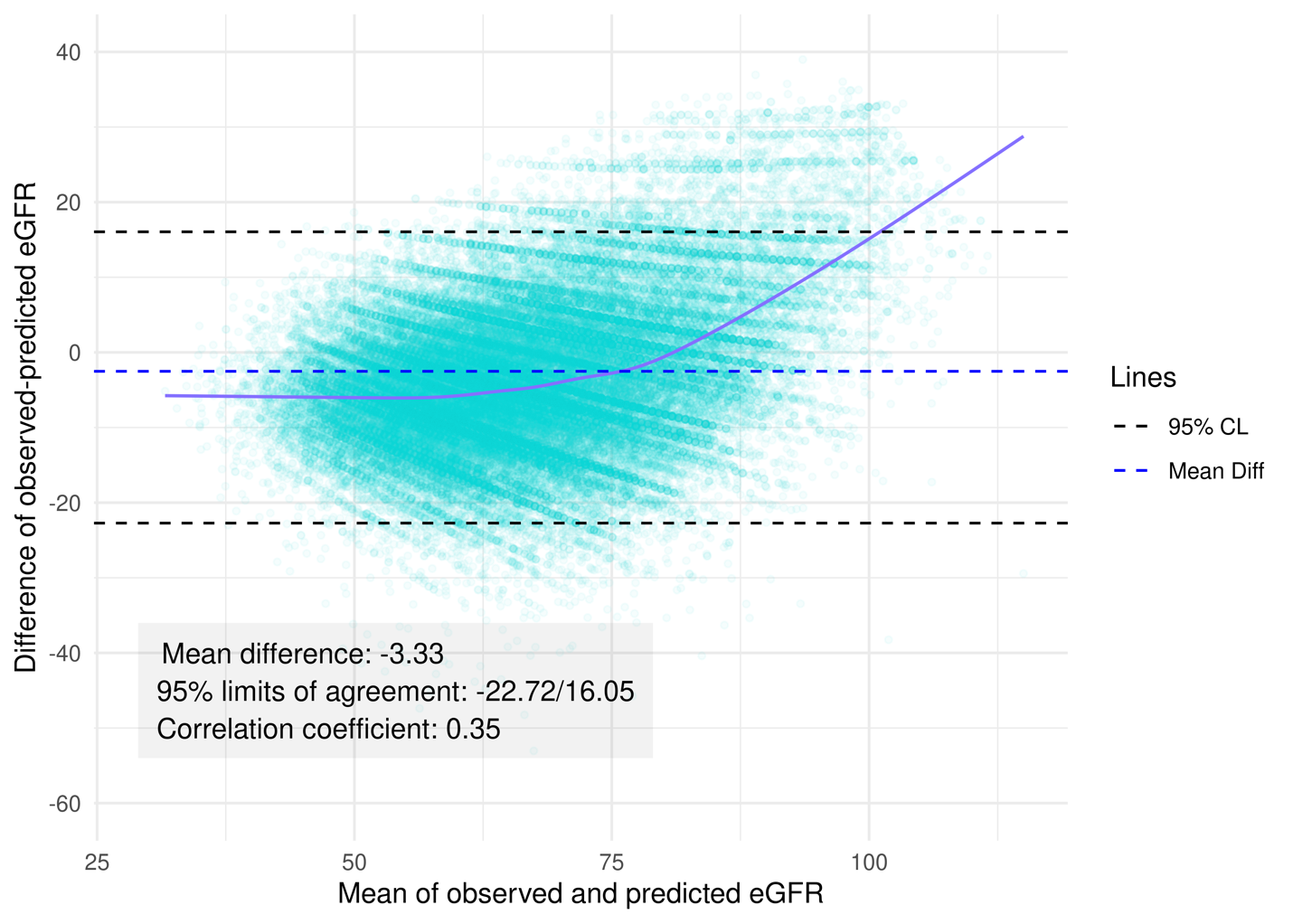
**

*eGFR12 = Postdonation 12-month estimated glomerular
